# Validation of a novel automated signal analysis tool for ablation of Wolff-Parkinson-White Syndrome

**DOI:** 10.1371/journal.pone.0217282

**Published:** 2019-06-26

**Authors:** Scott R. Ceresnak, Robert H. Pass, Anne M. Dubin, Lingyao Yang, Kara S. Motonaga, Haley Hedlin, Kishor Avasarala, Anthony Trela, Doff B. McElhinney, Christopher Janson, Lynn Nappo, Xuefeng B. Ling, Gregory J. Gates

**Affiliations:** 1 Stanford University—Lucile Packard Children’s Hospital, Division of Pediatric Cardiology, Pediatric Electrophysiology—Department of Pediatrics, Palo Alto, CA, United States; 2 The Children’s Hospital at Montefiore–Albert Einstein College of Medicine, Division of Pediatric Cardiology, Pediatric Electrophysiology—Department of Pediatrics, Bronx, NY, United States; 3 Stanford University–Department of Medicine, Quantitative Science Unit, Palo Alto, CA, United States; Universidad Miguel Hernandez de Elche, SPAIN

## Abstract

**Background:**

In previous pilot work we demonstrated that a novel automated signal analysis tool could accurately identify successful ablation sites during Wolff-Parkinson-White (WPW) ablation at a single center.

**Objective:**

We sought to validate and refine this signal analysis tool in a larger multi-center cohort of children with WPW.

**Methods:**

A retrospective review was performed of signal data from children with WPW who underwent ablation at two pediatric arrhythmia centers from 2008–2015. All patients with WPW ≤ 21 years who underwent invasive electrophysiology study and ablation with ablation signals available for review were included. Signals were excluded if temperature or power delivery was inadequate or lesion time was < 5 seconds. Ablation lesions were reviewed for each patient. Signals were classified as successful if there was loss of antegrade and retrograde accessory pathway (AP) conduction or unsuccessful if ablation did not eliminate AP conduction. Custom signal analysis software analyzed intracardiac electrograms for amplitudes, high and low frequency components, integrated area, and signal timing components to create a signal score. We validated the previously published signal score threshold 3.1 in this larger, more diverse cohort and explored additional scoring options. Logistic regression with lasso regularization using Youden’s index criterion and a cost-benefit criterion to identify thresholds was considered as a refinement to this score.

**Results:**

347 signals (141 successful, 206 unsuccessful) in 144 pts were analyzed [mean age 13.2 ± 3.9 years, 96 (67%) male, 66 (45%) left sided APs]. The software correctly identified the signals as successful or unsuccessful in 276/347 (80%) at a threshold of 3.1. The performance of other thresholds did not significantly improve the predictive ability. A signal score threshold of 3.1 provided the following diagnostic accuracy for distinguishing a successful from unsuccessful signal: sensitivity 83%, specificity 77%, PPV 71%, NPV 87%.

**Conclusions:**

An automated signal analysis software tool reliably distinguished successful versus unsuccessful ablation electrograms in children with WPW when validated in a large, diverse cohort. Refining the tools using an alternative threshold and statistical method did not improve the original signal score at a threshold of 3.1. This software was effective across two centers and multiple operators and may be an effective tool for ablation of WPW.

## Introduction

Wolff-Parkinson-White syndrome (WPW) affects 0.1 to 0.3% of all individuals and has become one of the most common indications for invasive electrophysiology study (EPS) and ablation in children [1–5]. Though invasive EPS and ablation has become standard of care for curative treatment of WPW in children, there still remains an ablation failure rate of 5–10%.[6–9] Acute failure of ablation is usually secondary to one of several factors: 1) inability to technically deliver energy to the anatomic substrate, 2) anatomic location of the accessory pathway that makes ablation problematic or perilous, or 3) an inability to identify the precise location of accessory pathway tissue.[10,11] While there have been significant advances in the technology used to deliver ablation lesions effectively in anatomically challenging locations, there has been little advancement in the ability to identify the exact location of the accessory pathway.

In 2012, we demonstrated in a pilot feasibility study of thirty patients with WPW that a novel, fully automated, computerized signal analysis software tool could retrospectively identify the site of successful ablation.[12] Based on the promising results of that study, we sought to expand and improve upon the pilot work and to determine if the novel signal analysis software could accurately predict the site of success from a larger, more diverse cohort of patients with WPW. Our primary aim was to determine if the software could work in a cohort of children with WPW and if this software could be refined by an alternative threshold or statistical method to enhance accuracy and predicative ability. In addition, we sought to determine how the software would perform in a variety of conditions including: differing catheters (RF vs cryocatheters), different EP laboratories, and different approach to the substrate (transseptal vs. retrograde). We also wanted to expand the use of this algorithm to congenital heart disease patients and to determine if the software could potentially provide a reduction in the number of lesions placed in those ablation cases that required more ablation attempts. We hypothesized that this signal analysis tool would have good predictive abilities under different conditions and could reliably distinguish successful from unsuccessful ablation sites in children with WPW across a range of technical conditions in a larger and more diverse cohort of patients.

## Methods

The study was a two-center retrospective review of patients with WPW undergoing invasive EPS and ablation at Lucile Packard Children’s Hospital—Stanford University and The Children’s Hospital at Montefiore–Albert Einstein College of Medicine. Institutional Review Board (IRB) approval was obtained at each institution for this investigation (Stanford University Research Compliance Office and IRB and the Children’s Hospital at Montefiore IRB), all data were fully anonymized and the IRB waived the requirement for informed consent for this retrospective study. Patients ≤ 21 years of age undergoing invasive EPS with attempted ablation between 2008 and 2015 were included. Patients with atrio-fasiciaular, nodo-ventricular, fasiculo-ventricular connections, or those patients with WPW requiring ventricular pacing for ablation were excluded. Signals were also excluded if there was poor power delivery (< 20 Watts), poor temperature delivery (< 40 degrees Centigrade), or lesion time less than 5 seconds, as we could not be certain that the etiology of the unsuccessful application was inadequate lesion formation versus signal quality. Data collected for this investigation included patient-specific demographic information, EPS study data, and the raw signal data from the EP laboratory during ablation.

### Signal analysis and novel signal analysis software

**Fig 1:**
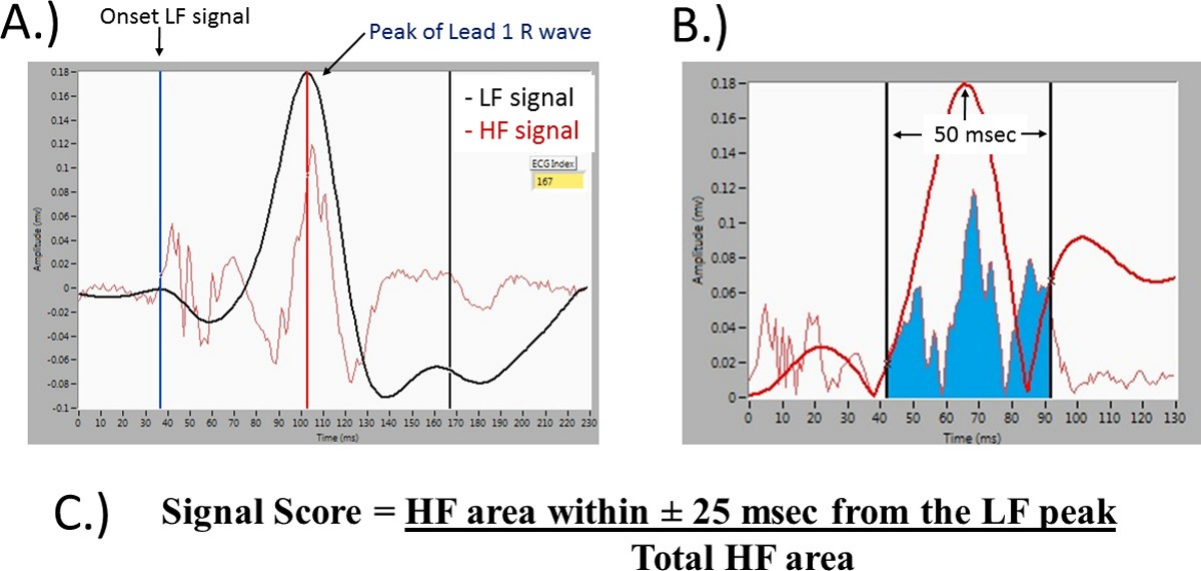
Novel signal analysis composite score (signal score). This figure demonstrates the generation of the composite Signal Score. A.) The high frequency (HF) and low frequency (LF) components of the signal on the distal ablation catheter are filtered and the onset of the LF and peak of the R wave on surface lead I are identified. B.) The area under the curve of the high frequency signal within a window of 50 msec, from 25 msec before to 25 msec after the peak of the LF signal, is calculated as the composite signal score (C.).

A detailed description of the signal analysis tool was provided in our prior pilot data publication.[12] In brief, all data were extracted from each patient from the GE CardioLab EP recording system at each institution (GE Healthcare, Piscatway, NJ). The raw data from the distal ablation probe were extracted as text data and exported from the recording system to a secure USB recording platform. Data were recorded with a standard high pass filter set at 30 Hz and a low pass filter set at 500 Hz. A *successful* signal was defined as a signal where ablation resulted in loss of AP conduction with sudden loss of pre-excitation. An unsuccessful signal was defined as a signal where an ablation attempt did not result in disruption of AP conduction with no change in the pattern of pre-excitation. The beat immediately prior to application of RF or cryoenergy was chosen for analysis. Each case was reviewed from start to finish by a pediatric electrophysiologist to ensure quality of the successful and unsuccessful signals. As described in the pilot manuscript, after extraction of the data, the signals were filtered with a standard Butterworth filter and divided into high frequency (HF) and low frequency (LF) components.[12] The signals were then analyzed by proprietary Labview (National Instruments, Austin, TX) software as previously described to automatically identify the following elements: the start of the HF signal, the peak of the LF signal, and the peak of the R wave on surface ECG lead I. The software was then able to calculate the following: the amplitude of the HF and LF signals (mV), the time from onset of the HF signal to the peak of the LF signal (msec), the time from the peak of the LF signal to the peak of the R wave in surface ECG lead I (msec), and the total area under the curve for the LF and HF signals. The predictive ability of each of these variables was re-assessed with this new, larger and more diverse cohort. Based on the pilot data, the best means of distinguishing a successful versus an unsuccessful signal was via a composite *signal score* that was calculated as the area under the curve of the HF signal with ± 25 msec of the peak of the LF signal (Fig 1). Pilot data suggested a threshold score of 3.1 to distinguish successful from unsuccessful signals and this threshold score and the other variables/thresholds were re-visited in this larger scale investigation. To determine if the signal processing tool could have helped reduce the number of lesions in those cases that required multiple ablation applications, a *difficult ablation* was defined as a procedure that required greater than the median number of lesions that was seen for the cohort as a whole.

### Data presentation and statistical analysis

Categorical and dichotomous variables are expressed as numbers and percentages whereas continuous variables are expressed as mean ± standard deviation or median (inter-quartile range) if not normally distributed. Continuous measurements from successful and unsuccessful signals were compared via repeated measures ANOVA, if normally distributed, and log transformed data if not normally distributed. Logistic regression with Lasso regularization, using the signal features calculated by the software as predictors, was considered as a refinement to the previous tool. In our logistic model, variables were determined a priori and we adjusted for gender, time between the onset of the high frequency signal and peak of the R wave on surface lead one (HF to R wave time), time between the peak of the high and low frequency signals (HF to LF time), time between the low frequency peak and peak of the R wave on surface lead I (LF to R wave time), congenital heart disease, location of the accessory pathway (left vs. right sided), integrated area under the curve of the low and high frequency signals (LF and HF integral values), and the area under the curve of the HF signal with ± 25 msec of the peak of the LF signal (Composite signal score). Data that was not normally distributed was log transformed. We then used the coefficients generated from Lasso model to calculate a predicted probability of success for each signal and then used the Youden’s index and a cost-benefit criterion to identify other potential thresholds for distinguishing successful from unsuccessful signals. The Youden's index (J), is the difference between the true positive rate and the false positive rate. Maximizing this index identifies, from the ROC curve, an optimal cut-off point independent of prevalence. The cost-benefit criterion allows us to choose a threshold that weights specificity more than sensitivity, depending on the size of the cost-benefit ratio we specify. We also considered alternative signal score thresholds besides 3.1, chosen using Youden’s index and cost-benefit ratio. Receiver operator curves (ROC) were generated to determine the predictive value of the signal analysis tool and the sensitivity and specificity produced from each statistical model were compared. To determine the potential benefit of the signal analysis tool in the *challenging* procedures, the median number of lesions for the entire cohort was identified and the percentage of lesions that would have been avoided with the signal analysis tool was determined by calculating the percentage of correct classifications in that cohort. Sub-group analysis of the predictive ability was also performed based on additional factors (such as accessory pathway location, presence of congenital heart disease, catheter type, ablation approach) and data are presented as percentage of signals that were classified correctly by the signal analysis tool. All two-sided *p*-values < 0.05 were considered statistically significant. Statistical analysis was performed using Stata (Stata Corp LP, College Station, TX, version 12) and R (R Core Team, Vienna, Austria, version 3.1.3). Data are provided as supplemental data files (S1 and S2 Files)

## Results

### Patient population

A total of 144 patients with WPW from both centers from 2008–2015 had signals available for review and were included in this analysis. A detailed description of the patient population and ablation-specific detail is demonstrated in Table 1. The majority of patients (141, 98%) underwent a single EPS, but 3 patients (2%) had a prior EPS and ablation attempt. The majority of patients underwent primary ablation with RF (94%). The RF ablation catheters that were used included: 76 Biosense Webster Navistar or EZ-Steer 4mm tip catheter, 48 Boston Scientific EPT 4mm tip catheter, 10 Mariner, 4 St Jude 5mm tip Saphyre. In the 8 patients undergoing cryoablation, 6 were with a 6 mm tip catheter, 1 with a 4mm tip catheter, and 1with an 8mm tip catheter.

**Table 1:** Patient population, EPS and ablation related data.

	Total Patients (n = 144)
Age (years)	13.9 ± 3.9
Weight (kg)	54 ± 27
Gender (n)	
Male	96 (67%)
Female	48 (33%)
Left sided AP (n)	66 (46%)
Transseptal approach (n)	44 (67%)
Retrograde approach (n)	22 (33%)
Multiple accessory pathways (n)	2 (0.1%)
Successful ablation (n)	140 (97%)
Ablation Type	
Radiofrequency (n)	136 (94%)
Cryo (n)	8 (6%)
Mean time to loss of AP conduction with ablation (sec)	
Time with RF	3.2 ± 2.6
Time with cryo	17.9 ± 4.1
Congenital Heart Disease (n)*	7 (5%)
Ablation with atrial pacing (n)	9 (6%)
3D electro-anatomic mapping system utilized (n)	90 (67%)
Procedural complications (n)	0 (0%)

*Patients with congenital heart disease included: Ebstein’s anomaly (2), TOF, d-TGA, ASD/PS, and severe LPA stenosis.

### Signal analysis data

**Fig 2:**
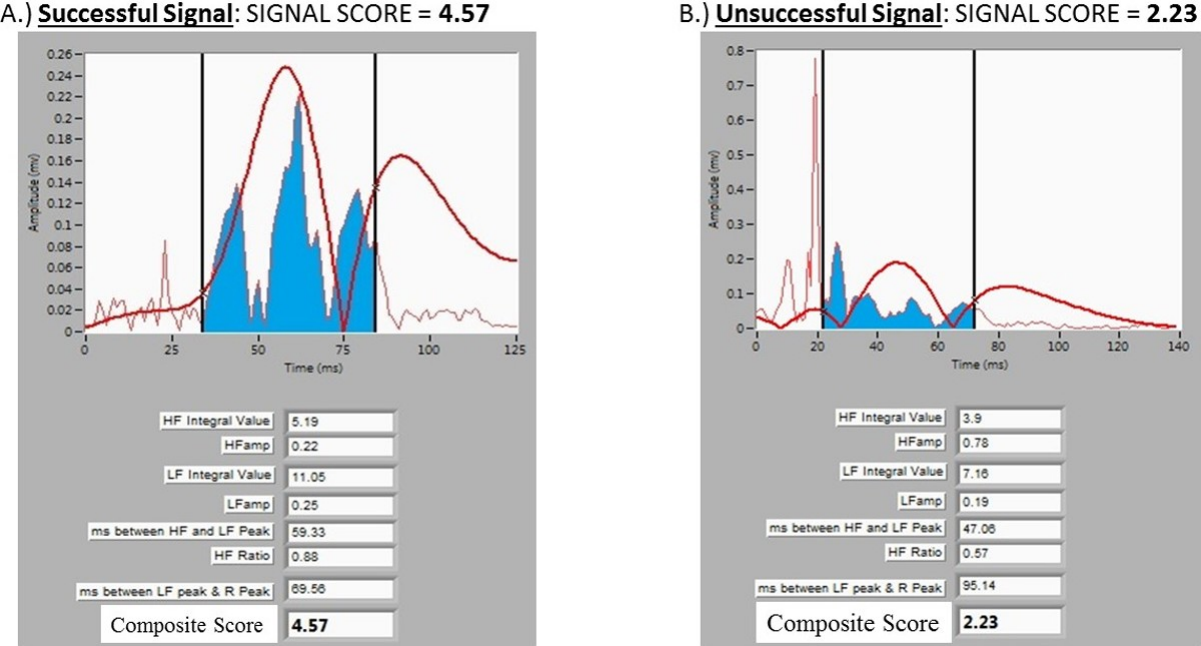
Example of successful and unsuccessful signals. (A) An example of a successful signal with a score 4.57. (B) An example of an unsuccessful signal in the same patient with a score of 2.23.

**Fig 3:**
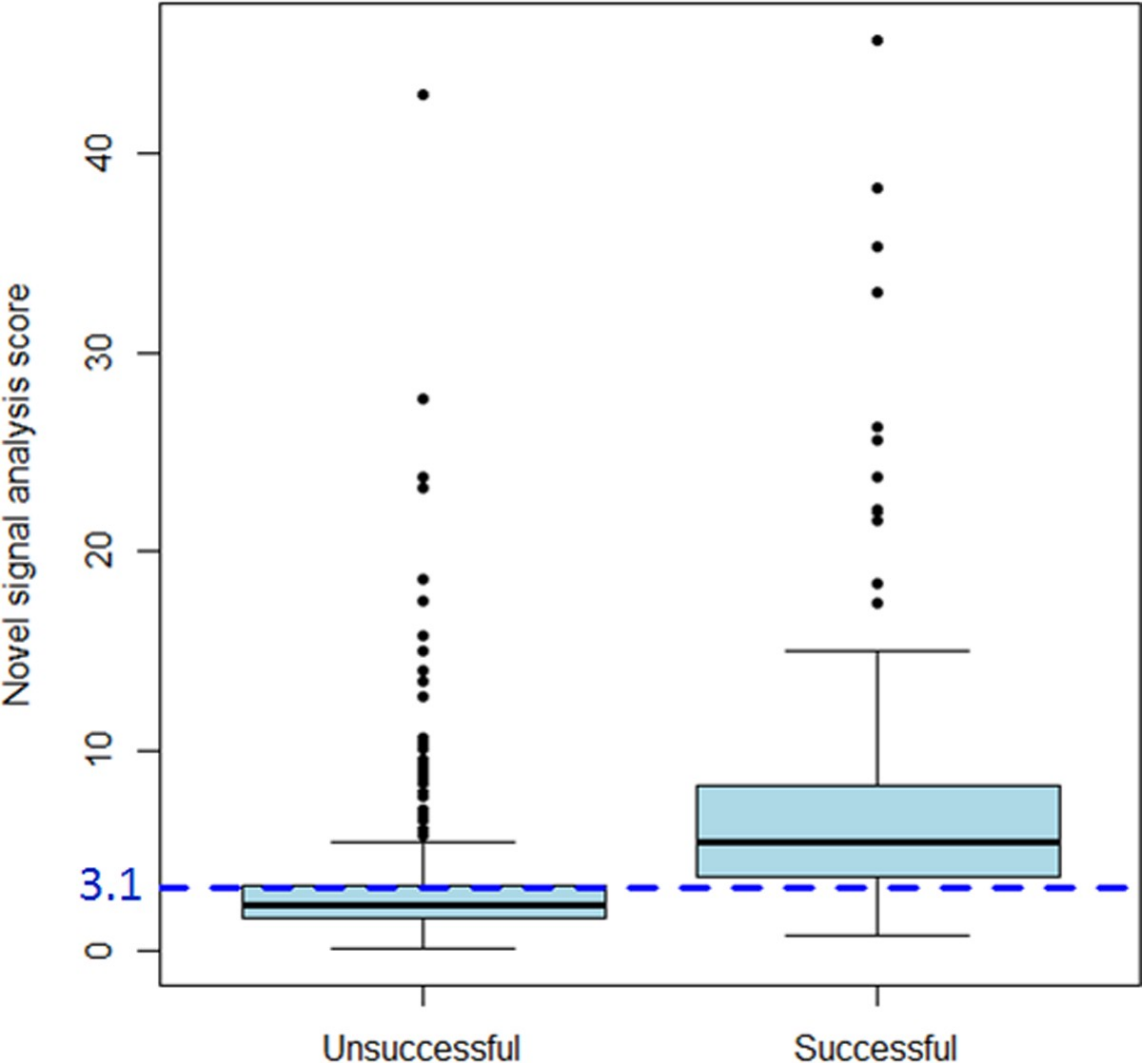
Box plot of successful vs. unsuccessful ablation signal scores. The figure demonstrates box plots of the composite signal score for successful and unsuccessful signals, with the threshold value of 3.1 highlighted with the dashed line. The bold line in the middle of the box represents the median, and the box defines the 25^th^-75^th^ percentiles (the interquartile range [IQR]). The lower whisker denotes the 25^th^ percentile– 1.5*IQR and the upper whisker denotes the 75^th^ percentile + 1.5*IQR. Observations outside of this range are plotted as dots.

**Fig 4:**
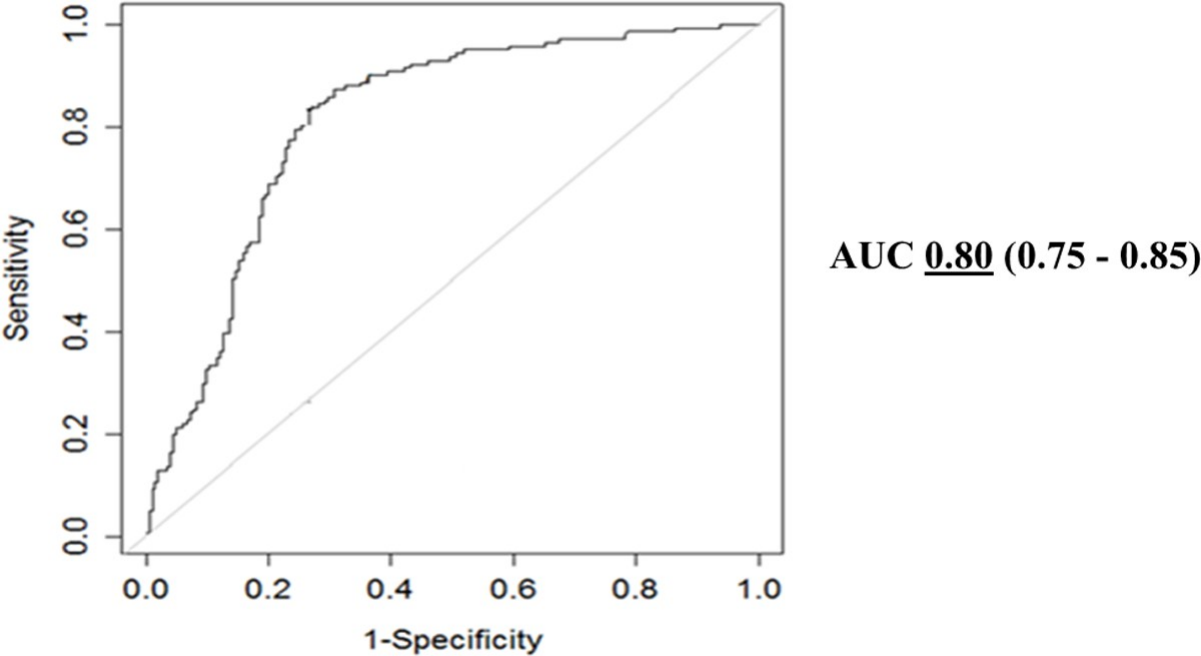
ROC curve of signal analysis software. A receiver-operator-curve of the signal analysis tool demonstrates an area-under-the-curve of 0.80.

**Fig 5:**
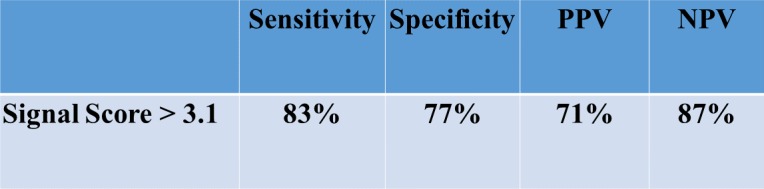
Predictive value of signal analysis tool. The figure demonstrates the sensitivity, specificity, positive and negative predictive values of the signal analysis tool. (PPV = positive predictive value, NPV = negative predictive ability).

In these 144 patients, a total of 347 signals were reviewed including 141 successful and 206 unsuccessful signals. The median number of lesions placed for each patient was 2 [Range 1–5] with the largest number of lesions placed in one patient being 23. A representative example of the software signal analysis output comparing a single patient’s successful and unsuccessful signals is shown in Fig 2A and 2B. A comparison of the signal analysis tool variables between successful and unsuccessful signals is demonstrated in Table 2. The logistic regression analysis revealed that the composite signal score identified from pilot data provided the best predictive ability with a higher sensitivity and specificity than the logistic regression approach and nearly the same area under the curve (AUC) (Table 3). Based on the cost-benefit ratio criterion, a threshold of 3.3 was compared to the composite score signal of 3.1 identified in pilot data and using Youden’s index. Given the similar specificity, sensitivity, and AUC, we determined the ideal threshold for the *signal score* based on the new cohort was identical to the prior work at 3.1 (Table 3). A box plot of the composite scores in successful versus unsuccessful signals is shown in Fig 3. Using this threshold of 3.1, the signal analysis software correctly predicted 274 of the 347 signals (80%) as either successful or unsuccessful. Successful signals were predicted correctly in 122 of 141 signals (87%), while unsuccessful signals were correctly predicted in 152 of 206 signals (73%). The receiver-operator-curve for a threshold signal score of 3.1 revealed an AUC of 0.80, indicating good predictive ability of the signal score in distinguishing a successful from an unsuccessful signal (Fig 4). The predictive ability of the signal score > 3.1 is shown in Table 3 and Fig 5.

**Table 2:** Novel signal analysis parameters data.

Variable	All signals (n = 347)	Successful Signals (n = 141)	Unsuccessful Signals (n = 206)	p-value*
HF Amplitude (mV)	0.39 (0.21–0.75)	0.53 (0.30–0.91)	0.31 (0.17–0.67)	< 0.001
LF Amplitude (mV)	0.23 (0.15–0.34)	0.30 (0.21–0.44)	0.19 (0.13–0.28)	< 0.001
HF to LF time (msec)	55 (44–68)	52 (44–62)	57 (45–70)	0.13
HF to R wave time (msec)	128 (107–143)	126 (107–143)	129 (109–143)	0.67
LF to R wave time (msec)	70 ± 24	71 ± 27	70 ± 22	0.81
Integral of HF signal	8.3 (5.8–13.8)	11.6 (7.5–16.2)	6.8 (5.1–11.3)	< 0.001
HF ratio	0.66 (0.52–0.79)	0.75 (0.62–0.84)	0.61 (0.47–0.73)	< 0.001
HF integral value	5.2 (3.5–8.8)	7.7 (5.8–10.9)	3.9 (2.9–5.8)	< 0.001
LF integral value	8.4 (5.4–12.4)	10.8 (6.8–16.1)	7.3 (4.5–10.6)	< 0.001
Composite signal score	3.1 (2.1–5.8)	5.4 (3.7–8.3)	2.3 (1.6–3.2)	< 0.001

*Comparison of successful to unsuccessful signals

Abbreviations: HF = high frequency, LF = low frequency, R wave = peak of the R wave in surface ECG lead I.

**Table 3:** Comparison with the original signal score and logistic regression with lasso regularization.

	Original score	Threshold refinement		Probability from Logistic Regression with Lasso regularization		
	Youden Index	Cost-Benefit Ratio = 1.5	Cost-Benefit Ratio = 2	Youden Index	Cost-Benefit Ratio = 1.5	Cost-Benefit Ratio = 2
**Threshold**	3.1	3.3	3.3	0.34	0.46	0.53
**Sensitivity**	0.87	0.83	0.83	0.84	0.65	0.56
**Specificity**	0.74	0.77	0.77	0.73	0.82	0.85
**PPV**	0.70	0.71	0.71	0.68	0.71	0.72
**NPV**	0.89	0.87	0.87	0.87	0.77	0.74
**Area under** **the curve**	0.81 (0.77,0.86)	0.81 (0.77,0.86)		0.81 (0.76,0.87)		

**Fig 6:**
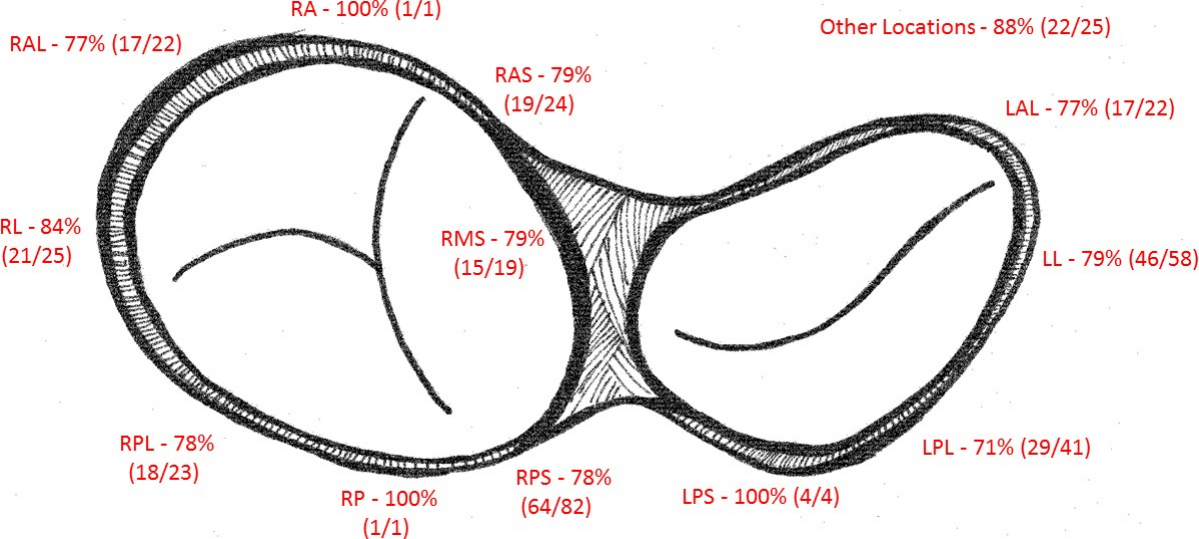
Predictive ability by accessory pathway location. The figure demonstrates the predictive ability of the signal analysis tool by accessory pathway location on the tricuspid and mitral annuli. The numbers next to the accessory pathway location indicate the percentage of signals in that location that were classified correctly and the numbers in parenthesis indicated the number correctly classified signals over the total number of signals in that location. (LAL = left antero-lateral, LL = left lateral, LPL = left postero-lateral, LPS = left postero-septal, RA = right anterior, RAL = right antero-lateral, RAS = right antero-septal, RMS = right mid-septal, RL = right lateral, RP = right posterior, RPL = right poster-lateral, other locations include middle cardiac vein, CS diverticulum, and right atrial appendage).

We also analyzed the ability of the composite score to correctly classify signals in certain subgroups and with certain ablation substrates. There was no difference in the signal score’s ability to correctly classify based on accessory pathway location (right sided 80% vs. left sided 77%, p = 0.50), presence of congenital heart disease (CHD 100% vs. no CHD 79%, p = 0.17), RF versus ablation catheter (RF 79% vs. Cryo 83%, p = 0.70), type of RF ablation catheter (Boston Scientific EPT 79% vs. Biosense Navistar 77% vs. Mariner 86%, p = 0.81), need for atrial pacing during ablation (APACED 81% vs. No Pacing 79%, p = 0.82), or ablation approach (transseptal 81% vs. retrograde 79%, p = 0.91). When looking at ablation location in more detail by dividing the locations into 13 discrete locations on each AV ring, there were no significant differences based on discrete locations. When analyzing locations with 10 or more signals available for review, the worst classification was noted in the left postero-lateral region (41 signals, 71% correctly classified as successful or unsuccessful) and the best classification noted in the right lateral region (22 signals, 84% correctly classified as successful or unsuccessful) [Fig 6]. Finally, the datasets were from pediatric EP laboratories, but the patient population included 14 patients over 18 (10%) and 40 pages over 16 (28%). There was no difference in the percentage signals classified correctly between the older patients the and the younger patients (< 16 80% vs. ≥ 16 74%; p = 0.28), suggesting the signal analysis software would work just as well in older patients or adults.

### Challenging ablations

To determine the potential ability of the signal analysis tool to limit the number of lesions during an ablation, we then analyzed the signals in patients that required a larger number of ablation lesions. As noted previously, the median number of lesions per patient was 2. All signals from patients requiring 3 or more ablation lesions were then analyzed to evaluate the performance of the 3.1 threshold in these challenging patients. There were 33 patients requiring 3 or more lesions with 209 signals analyzed from this group (31 successful, 178 unsuccessful). A total of 163 of the 209 signals (78%) were classified correctly, with 135 of the 178 unsuccessful signals (76%) also classified correctly.

## Discussion

Developments in automation have been able to leverage the power of computer processing systems to augment the abilities of the bedside physician in a broad array of medical specialties. Technological growth has dramatically enhanced the practice of electrophysiology and there have been numerous attempts to improve the EP study and ablation process over the past 20 years. The development of RF and cryoablation, the use of automated computer based EP recording systems, and the development of 3-dimensional mapping systems to map and visualize the ablation substrate are examples that have enhanced the technical capabilities of the electrophysiologist.[13–17] While this tremendous progress has revolutionized the ablation process, there has been only minimal work attempting to harness the power of computational systems in signal analytics. In this study, we demonstrate that automated signal analysis tools can provide reasonable diagnosis accuracy for distinguishing the site of a successful ablation in children with WPW. This work demonstrates that the armamentarium of tools available for ablation of WPW may expand to the realm of automated signal analysis and automation of signal analytics may provide an additional tool in the EP laboratory.

The experienced electrophysiologist can often recognize the signal on the ablation catheter that will result in disruption of AP conduction. Standard measurement tools and operator experience in evaluating signals in the EP laboratory have proven powerful tools in the ablation of accessory pathways, but we can now harness the processing power of modern computational systems to augment and improve upon the current methodologies. Eliciting the assistance of computer technology to aid in ablation has the potential to help transform the manner by which electrophysiologists approach the analysis of electrical signals in the EP laboratory. In this series, we demonstrated that this software tool can identify sites of successful ablation with 83% sensitivity and 77% specificity with a positive predictive valve of 71% and negative predictive value of 87%. In real time, the ability to export a signal and then process and analyze the signal typically takes less than 1 minute. This signal analysis tool may thus potentially supplement the talents of the experienced electrophysiologist and add to the diagnostic abilities of the inexperienced operator.[18]

Though the success rates for WPW ablation are generally good, there is always value in improving our technical abilities and success rates while minimizing the number of lesions placed during ablation. There are numerous possible causes for unsuccessful ablation, including operator experience and technical ability, inadequate temperature or power delivery during ablation attempts, poor tissue contact limiting the ability to achieve tissue destruction, location of the accessory pathway (ie near the compact AV node or epicardial location), and poor or inappropriate analysis of the intracardiac signals.[10,11] There have been several therapeutic advances over the past two decades to help address some of these potential factors, including development of cryoenergy to ablate near the compact AV node, three-dimensional anatomic mapping systems, and, more recently, contact force catheters.[13–16] There have been few advances over the past 15 years, however, that have significantly altered the landscape of signal analysis to enhance the ablation process for children undergoing ablation. While there may be some ability of this tool to improve the overall success rates with ablation, perhaps the greatest improvement in the ablation process may be derived by decreasing the number of lesions needed for a successful ablation.

In addition to decreasing the number of unnecessary ablation lesion, one of the potential benefits of this tool may be related to the strong predictive ability in the right lateral region of the tricuspid annulus. Ablation success rates have been demonstrated to be particularly low in the right lateral region, with the PAPCA data demonstrating a success rate in the right lateral area as low as 85% and a high recurrence rate of up to 16%.[6,10,19,20] The predictive ability of our signal analysis tool was particularly good in the right lateral region, correctly identifying the site of a successful ablation in 84% of right lateral signals. While tissue contact, experience, and identifying an appropriate annular position are all likely factors in this lower success rate, perhaps use of a signal analysis tool such as this can also improve success rates in this particularly challenging location.[10,11]

Our initial pilot work consisted of 30 patients with only 15 patients in which we could compare successful to unsuccessful ablation electrograms. The pilot work provided proof of concept, but the population analyzed was limited in scope. All patients from that pilot study were from one EP laboratory and all ablations signals were using the same ablation catheter type. Patients who underwent cryoablation or who were atrially paced were excluded and all left-sided pathways were approached from a transseptal approach. In this analysis, we included two distinct EP laboratories and numerous different ablation catheters, patients who underwent cryoablation, patients who were atrially paced during ablation, patients with congenital heart disease, and patients who underwent ablation of a left-sided pathway via a retrograde approach. Though numbers of some of these sub-groups were small, the signal processing tool performed similarly across all of these variables. The software thus had the ability to translate across centers and with different ablation tools, operators and approaches.

Further prospective study of this tool is warranted to verify that the retrospective predictive ability translates into real-time enhancement of ablation of WPW. Automated signal analysis strategies may thus herald a new era of signal analysis that incorporates the integration of computational analytics to the ablation of anatomic substrates.

### Limitations

There are several limitations to this study. All data extraction for this investigation occurred via the GE Cardiolab recording systems. Applicability of this software to other recording systems requires further investigation. In addition, contact force catheters were not utilized during this time and it is possible that an ablation was unsuccessful at a particular site because tissue contact was poor and not solely due to poor signal analysis. The overall number of patients with congenital heart disease, atrial pacing, multiple accessory pathways and ablation using cryoenergy were small. While the signal analysis software provided a similar degree of diagnostic accuracy to other ablation groups and strategies, interpretation of the data in these select sub-groups will be limited. For this signal analysis software only one beat is required for analysis and a beat without significant noise or artifact should be ideally chosen. Finally, different labs could have slightly different configurations and prior to using this software each individual lab would have to link the output variables from the GE system to the input variables on the novel signals software.

## Conclusions

An automated signal analysis software tool reliably distinguished electrograms at sites that resulted in acutely successful ablation of WPW from unsuccessful ones. This software was effective across two centers, multiple operators, and different ablation catheters. This signal analysis software may be an effective tool for aid in the ablation of WPW in children.

## Supporting information

S1 File Supporting supplemental data set. (XLS)

S2 File Supporting supplemental data set in long form. (XLS)

## Abbreviations

AP: Accessory pathway
AVRT: Atrio-ventricular reciprocating tachycardia
EP: Electrophysiology
EPS: Electrophysiology study
Cryo: Cryoenergy
HF: High frequency
LF: Low frequency
RF: Radiofrequency energy
SVT: Supraventricular tachycardia
WPW: Wolff-Parkinson-White Syndrome
